# Voltage-Gated Sodium Channels in Neocortical Pyramidal Neurons Display Cole-Moore Activation Kinetics

**DOI:** 10.3389/fncel.2018.00187

**Published:** 2018-06-26

**Authors:** Mara Almog, Tal Barkai, Angelika Lampert, Alon Korngreen

**Affiliations:** ^1^The Mina and Everard Goodman Faculty of Life Sciences, Bar Ilan University, Ramat Gan, Israel; ^2^The Leslie and Susan Gonda Interdisciplinary Brain Research Center, Bar Ilan University, Ramat Gan, Israel; ^3^Institute of Physiology, RWTH Aachen University, Aachen, Germany

**Keywords:** voltage-gated, sodium, channel, activation, model, kinetics

## Abstract

Exploring the properties of action potentials is a crucial step toward a better understanding of the computational properties of single neurons and neural networks. The voltage-gated sodium channel is a key player in action potential generation. A comprehensive grasp of the gating mechanism of this channel can shed light on the biophysics of action potential generation. However, most models of voltage-gated sodium channels assume a concerted Hodgkin and Huxley kinetic gating scheme. However, it is not clear if Hodgkin and Huxley models are suitable for use in action potential simulations of central nervous system neurons. To resolve this, we investigated the activation kinetics of voltage-gated sodium channels. Here we performed high resolution voltage-clamp experiments from nucleated patches extracted from the soma of layer 5 (L5) cortical pyramidal neurons in rat brain slices. We show that the gating mechanism does not follow traditional Hodgkin and Huxley kinetics and that much of the channel voltage-dependence is probably due to rapid closed-closed transitions that lead to substantial onset latency reminiscent of the Cole-Moore effect observed in voltage-gated potassium conductances. Thus, the classical Hodgkin and Huxley description of sodium channel kinetics may be unsuitable for modeling the physiological role of this channel. Furthermore, our results reconcile between apparently contradicting studies sodium channel activation. Our findings may have key implications for the role of sodium channels in synaptic integration and action potential generation.

## Introduction

Detailed mechanistic understanding of the action potential is a paramount goal in neuroscience. One major milestone dates back more than 60 years when Hodgkin and Huxley (Hodgkin and Huxley, 1952) showed that action potential generation required a fine balance between the fluxes of sodium and potassium ions through voltage-gated conductances. From then onwards, their kinetic model has served as a cornerstone for understanding the generation and propagation of action potentials in many systems. However, a much more detailed picture of the mechanisms underlying membrane excitation has emerged over the years that challenges several features of the Hodgkin and Huxley model (Armstrong and Bezanilla, 1977; Bezanilla and Armstrong, 1977; Raman and Bean, 1997, 2001). In contrast to physiologically oriented modeling, structure function investigations of voltage-gated channels usually describe channel kinetics using Markov chain models (Sakmann and Neher, 1995). These structure function studies have shown, for almost all voltage-gated ion channels, that Hodgkin and Huxley-like kinetics are only a specific approximation of the kinetic behavior of the channels (Patlak, 1991). Moreover, channel activation kinetics defines encoding properties of neuronal populations, and models of neurons with conventional Hodgkin and Huxley-like activation do not capture the full ability of neurons to encode high frequency and fast changing inputs.

Two prime deviations from the classical Hodgkin and Huxley kinetics have been observed in neurons from the rodent CNS. The voltage-gated channels in many CNS neurons display persistent currents and together with resurgent voltage-gated sodium currents greatly increase the contribution of sodium conductance to the action potential (Stuart and Sakmann, 1995; Fleidervish and Gutnick, 1996; Fleidervish et al., 1996; Raman and Bean, 1997, 2001; Raman et al., 1997; Brumberg et al., 2000; Astman et al., 2006; Kole and Stuart, 2008; Kole et al., 2008; Lewis and Raman, 2014). A number of studies have reported that the sodium channel activates with a longer delay than is predicted by the Hodgkin and Huxley model (Martina and Jonas, 1997; Schmidt-Hieber and Bischofberger, 2010). Interestingly, one study, focusing on voltage-gated sodium channel activation reported that the channels activate without delay (Baranauskas and Martina, 2006).

Thus, the kinetics of voltage-gated sodium channels deviate from the classical Hodgkin and Huxley model. However, there is disagreement how these kinetics should be formally expressed. To investigate this deviation and its potential impact on sodium channel activation, we conducted voltage-clamp experiments primarily from nucleated patches extracted from the soma of layer 5 (L5) pyramidal neurons. We show that the gating mechanism does not follow traditional Hodgkin and Huxley kinetics. Specifically, much of the channel voltage-dependence is due to rapid closed-closed transitions leading to substantial onset latency reminiscent of the Cole-Moore effect observed in voltage-gated potassium conductances (Cole and Moore, 1960).

## Methods

### Animals

All procedures were approved and supervised by the Bar-Ilan university Animal Care and Use Committee and were in accordance with the National Institutes of Health Guide for the Care and Use of Laboratory Animals and the University's Guidelines for the Use and Care of Laboratory Animals in Research. This study was approved by the National Committee for Experiments in Laboratory Animals at the Ministry of Health.

### Slice preparation

Slices (sagittal, 300 μm thick) were prepared from the somatosensory cortex of 12–16 days old Wistar rats that were killed by rapid decapitation using previously described techniques (Stuart et al., 1993). Slices were perfused throughout the experiment with an oxygenated artificial cerebrospinal fluid (ACSF) containing (mM): 125 NaCl, 25 NaHCO_3_, 2.5 KCl, 1.25 NaH_2_PO_4_, 1 MgCl_2_, 2 CaCl_2_, 25 Glucose, 0.5 Ascorbate (pH 7.4 with 5% CO_2_, 310 mosmol/kg). All experiments were carried out at room temperature (25°C). Pyramidal neurons from L5 in the somatosensory cortex were visually identified using infrared differential interference contrast (IR-DIC) videomicroscopy (Stuart et al., 1993).

### Electrophysiology

Nucleated outside-out patches (Sather et al., 1992; Korngreen and Sakmann, 2000; Almog and Korngreen, 2009) were extracted from the soma of L5 pyramidal neurons from the somatosensory cortex of P14 rats. Briefly, negative pressure (180–230 mbar) was applied when recording in the whole cell configuration, and the pipette was slowly retracted. Provided that the retraction was gentle it was possible to obtain large patches of membrane engulfing the nucleus of the neuron. After the extraction of the patch, the pressure was reduced to 30–40 mbar for the duration of the experiment. All measurements from nucleated patches were carried out with the Axopatch-700B amplifier (Axon Instruments, Foster City, CA) using a sampling frequency of 100 kHz and filtered at 20 kHz. The capacitive compensation circuit of the amplifier was used to reduce capacitate transients. Nucleated patches were held at −60 mV. Leak was subtracted using a P/4 online protocol. Patch pipettes (4–7 MΩ) were coated with Sylgard (DOW Corning). To record sodium currents in nucleated patches the pipettes were filled with a solution containing 120 mM Cs-gluconate, 20 mM CsCl, 10 mM HEPES, 4 mM MgATP, 10 mM phosphocreatine, 1 mM EGTA, 0.3 mM GTP (pH = 7.2, CsOH). The liquid junction potential of −11 mV generated by this pipette solution was not corrected for during the experiments or offline analysis. Cell attached recordings were performed from the soma of L5 pyramidal neurons using standard procedures (Hamill et al., 1981; Stuart et al., 1993). To record sodium currents in the cell attached mode the pipettes were filled with a solution containing 130 mM NaCl, 3 mM KCl, 2 mM MgCl_2_, 2 mM CaCl_2_, 10 mM HEPES, 4 mM TEA, 1 mM 4-aminopyridine, and 10 mM glucose (pH = 7.3 with HCl). Cell attached patches were held at −20 mV relative to the membrane potential. Similar to the nucleated patches, the pipettes were coated with Sylgard and the leak was subtracted using a P/4 online protocol. All cell-attached recordings were performed using the AxoPatch-200B amplifier (Axon Instruments, Foster City, CA) using a sampling frequency of 100 kHz and filtered at 20 kHz.

### Data analysis and numerical simulations

All off-line data analyses including curve fitting were carried out with IGOR (WaveMetrics, Lake Oswego, USA). Experimental results were observed in cells from two or more animals. Therefore, all the results from a specific protocol were pooled and displayed as means±S.E.M. Simulations of ionic currents were programmed using NEURON 7.3 (Hines and Carnevale, 1997). All simulations were performed with an integration interval of 5 μs to ensure a stable numerical solution of the differential equations. Ion channel models were implemented using the NMODL extension of NEURON (Hines and Carnevale, 2000). The models of channels reported in the literature were downloaded from ModelDB (https://senselab.med.yale.edu/ModelDB/showmodel.cshtml?model=128079 and https://senselab.med.yale.edu/ModelDB/showmodel.cshtml?model=62673) and used without any changes to the code.

## Results

**Figure 1:**
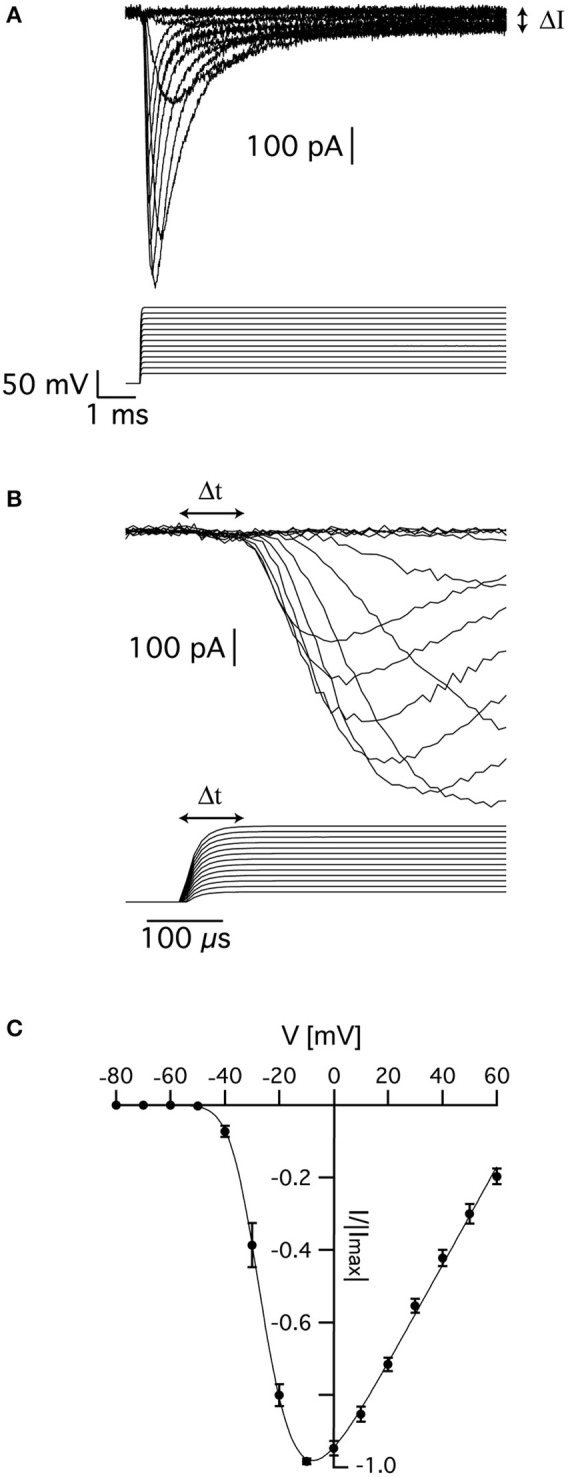
Voltage-gated sodium currents recorded in nucleated patches from cortical pyramidal neurons. **(A)** Representative recording of sodium currents from layer 5 neocortical pyramidal neurons. **(B)** Magnification of the time immediately following the voltage change showing the delayed activation of the sodium current. The vertical scale of the command potential is identical to the one presented in **(A)**. **(C)** activation curve calculated from 9 nucleated patches. The current in each patch was normalized to the absolute value of the maximal current amplitude recorded at −10 mV to reduce variability between patches. The line connecting the points is the curve fit of Equation (1). Error bars are SEM.

Sodium currents were recorded from nucleated patches after blocking voltage-gated potassium currents as detailed in the Methods. Fast inward currents could easily be detected following a depolarizing voltage clamp step from a holding potential of −110 mV (Figure 1A). We only analyzed currents from which the linear leak was cleanly subtracted by the P/4 protocol, thus leaving no residual of the capacitance transient. This was quite straightforward when the capacitance transient did not saturate the amplifier or exceed the dynamic range of the A/D converter. Due to the small membrane surface the total capacitance of a nucleated patch was small and allowed for excellent temporal control of the voltage-clamp amplifier (Martina and Jonas, 1997; Baranauskas and Martina, 2006). We verified this by measuring the decay of the uncompensated capacitance transients that decayed to baseline in under 40 μs. To be on the safe side, we removed the first 50 μs from the data before analysis. Note that this may result in an under- estimation of the channel kinetics.

As described elsewhere, the recorded currents displayed two features deviating from the classical description of voltage-gated sodium channels in that there was a sustained current (denoted by ΔI in Figure 1A) and delayed activation (denoted by Δt in Figure 1B). We first estimated the apparent voltage-dependence of channel activation by measuring the maximal current change in 9 patches. To reduce inter-patch variability, we normalized each patch to the maximal current obtained in this analysis. Plotting the maximal normalized current from 9 patches as a function of voltage produced a typical activation curve of voltage-gated sodium channels (Figure 1C). Fitting this curve with:

(1) $$\begin{array}{r} \frac{I}{\left|I_{max}\right|}=\frac{B*\left(V-E_{Na}\right)}{1+e^{\left(-\frac{V-V_{0.5}}{z}\right)}} \end{array}$$

where *B* is a proportion constant, *E*_*na*_ is the sodium reversal potential, *V*_0.5_ is the voltage of half activation, and *z* is the slope of the Boltzmann curve, we obtained V_0.5_ = −24 ± 2 mV, *z* = 5.2 ± 0.7 mV, and E_Na_ = 75 ± 5 mV (*n* = 9).

In the current study, we focused on the analysis of the delay in channel activation. The activation curve (Figure 1C) suggested that above −10 mV, the channel would approach its maximal open probability. Thus, in the simple scheme for the activation (disregarding inactivation) of the channel suggested below it was reasonable to assume that for depolarized potentials all backward rate constants would be much smaller than the forward rate constants and could thus be neglected.

In this voltage range, the average delay between the onset of the voltage command and reaching the open state is given by:

(2) $$\begin{array}{r} \delta =\sum_{i=1}^{N}\frac{1}{\alpha_{i}} \end{array}$$

**Figure 2:**
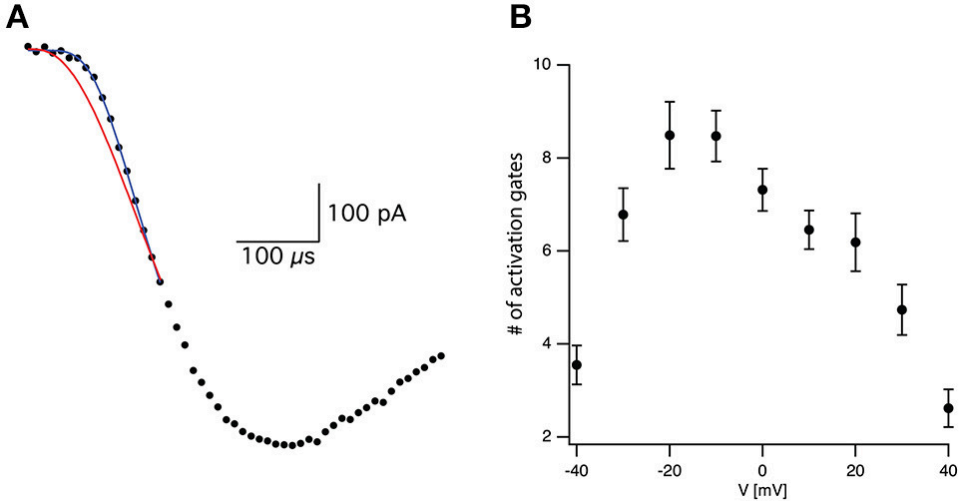
Estimating activation sigmoidicity for the voltage-gated sodium channel. **(A)** Representative curve fit of Equation (3) to the onset of current activation (black line). The current was recorded after a voltage step from −110 to −10 mV. The red line is a fit of the same data to Equation (3) in which the number of activation gates was set to 3 as in the Hodgkin and Huxley model. The black line is the fit of Equation (3) using the number of activation gates as a free parameter. This specific fit generated a value of *N* = 9 ± 1. **(B)** The apparent number of activation gates, obtained by fitting Equation (3) to data from 9 patches, is plotted as a function of the membrane potential. In all cases the patch was held at −110 mV for 150 ms prior to stepping to the test potential. Error bars are SEM.

**Scheme 1:**
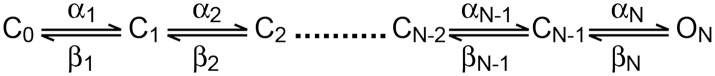
Linear activation scheme for a voltage-gated ion channel.

Assuming that the forward rate constants increase exponentially with voltage it is simple to show that δ will decrease as a function of voltage. Clearly, δ is dependent on the number of transitions leading to channel opening and the overall rate of the activation process. It is less sensitive to interdependence between rate constants in the activation scheme. The expression for δ in a sequential model only differs from a concerted model by a proportionality constant. Together, the number of transitions and rate of activation defined the amount of delay relative to the overall rate of activation or sigmoidicity of the activation process (Zagotta et al., 1994a,b; Schoppa and Sigworth, 1998a,b). In voltage-gated potassium channels the sigmoidicity was estimated by fitting an exponential function to the slowest part of the current trace followed by extrapolation to a zero current providing an estimate for δ (Zagotta et al., 1994b; Schoppa and Sigworth, 1998b). In the current study, this was obviously not possible due to channel inactivation. Thus, we assumed that the initial stages of the activation were only marginally affected by inactivation. This assumption was put to the test at a later stage (see **Figure 4**). Thus, we only analyzed the current up to 50% of its maximal current by fitting it with:

(3) $$\begin{array}{r} I=I_{max}{\left(1-e^{-t/\tau }\right)}^{N} \end{array}$$

where τ is the time constant and N is the apparent number of transitions in the Markov chain model (Figure 2A). Equation (3) is not the general solution to Scheme 1 but rather the concerted version of that model. Using it considerably reduced the number of free parameters to be fitted. Previously it was shown that fitting this equation produces practically indistinguishable results from the general solution to Scheme 1 (Zagotta et al., 1994b; Schoppa and Sigworth, 1998b) due to the difficulty of distinguishing between certain kinetic models using whole-cell currents (Milescu et al., 2005).

Fitting Equation (3) to the current onset with *N* = 3 as in the Hodgkin and Huxley model (red line in Figure 2A) failed to provide a complete fit for the data. Fitting the current onset this time using N as a free parameter produced an improved fit with *N* = 9.1 for the trace displayed in Figure 2A (black line in Figure 2A). As predicted by Equation (2), the apparent number of closed states signifying the sigmoidicity of the current trace decreased as a function of voltage above −10 mV (Figure 2B). We extended the fit to lower potentials that displayed smaller sigmoidicity probably due to the participation of backward transitions slowing the current trace. Thus, our phenomenological analysis supported a model for the activation of voltage-gated sodium channels in L5 pyramidal neurons containing considerably more closed states than the three gates assumed by the Hodgkin and Huxley model.

Early results for the voltage-gated potassium channel of the giant squid axon indicated that holding potential could modulate latency before the onset of activation (Cole and Moore, 1960). Hyperpolarization of the membrane potential increased the latency of current activation. These authors reported that these effects increased the apparent number of activation gates in the potassium channel from 4 to 25. This may suggest that the prolonged delay and steep current activation we observed in our recordings from voltage-gated sodium channels were due to a “Cole-Moore effect.”

**Figure 3:**
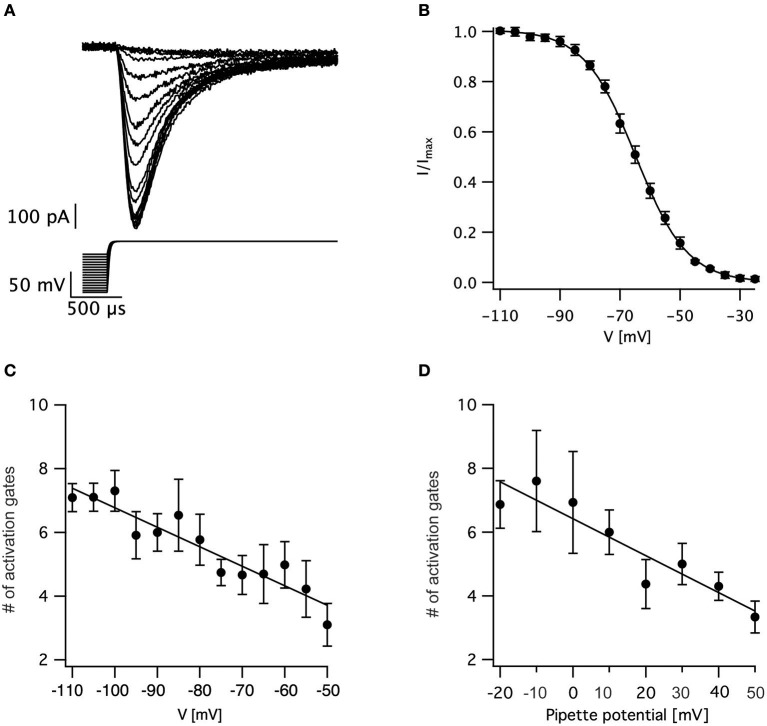
Estimating the voltage-dependence of activation sigmoidicity in the channel inactivation experiment. **(A)** representative recording of currents evoked by a voltage-clamp protocol designed to measure steady-state inactivation. The 150 ms pre-pulse to various potentials was truncated to facilitate the display of the current. In all trials, the voltage was stepped to 0 mV following the pre-pulse. **(B)** Steady-state inactivation curve calculated from 11 recordings identical to those displayed in **(A)**. The current was normalized to the trace obtained following a pre-pulse to −110 mV. The smooth line of a fit of a Boltzmann function. **(C)** The apparent number of activation gates, obtained by fitting Equation (3) to data from 9 nucleated patches, plotted as a function of the pre-pulse potential. **(D)** The apparent number of activation gates, obtained by fitting Equation (3) to data from 3 cell-attached patches, plotted as a function of the pre-pulse potential which is displayed as the pipette potential since the true membrane potential is unknown. Error bars in are SEM in all subfigures.

We tested this prediction by varying the holding potential prior to channel activation. Using a standard protocol designed for measuring the steady-state inactivation of voltage-gated sodium channels we varied the holding potential from −110 to −25 mV for a period of 150 ms prior to stepping the potential to zero mV to record the current (Figure 3A). The maximal current change following each pre-pulse was normalized to the maximal current change recorded following the pre-pulse to −110 mV. The resulting normalized inactivation curve, averaged across patches, is presented in Figure 3B. Fitting this curve to a Bolzmann function gave V_0.5_ = −64.5 ± 0.5 mV with *z* = 8.3 ± 0.3 mV (*n* = 11). Next, we fitted the first 50% rise of the current to Equation (3). As predicted, for a sequential activation model containing several closed states, the apparent number of activation gates decreased steadily as the pre-pulse potential increased (Figure 3C) which is consistent with results obtained for the sodium channel in the squid giant axon (Keynes and Rojas, 1976). This supports the hypothesis that the activation of voltage-gated sodium channels in L5 pyramidal neurons displayed Cole-Moore kinetics, probably due to many sequential closed transitions before the open state. However, at this stage we could not dismiss the possibility that these results might be an experimental artifact related to the extraction of the nucleated outside-out patch. Thus, we also recorded sodium currents in the cell-attached mode of the patch-clamp technique and subjected them to the same analysis. Out of the 78 patches only three had large enough currents for the analysis of current onset. Estimating the apparent number of activation gates from these patches (Figure 3D) showed a similar dependence of the apparent number of gates on the command potential to that obtained from nucleated patches (Figure 3C).

**Figure 4:**
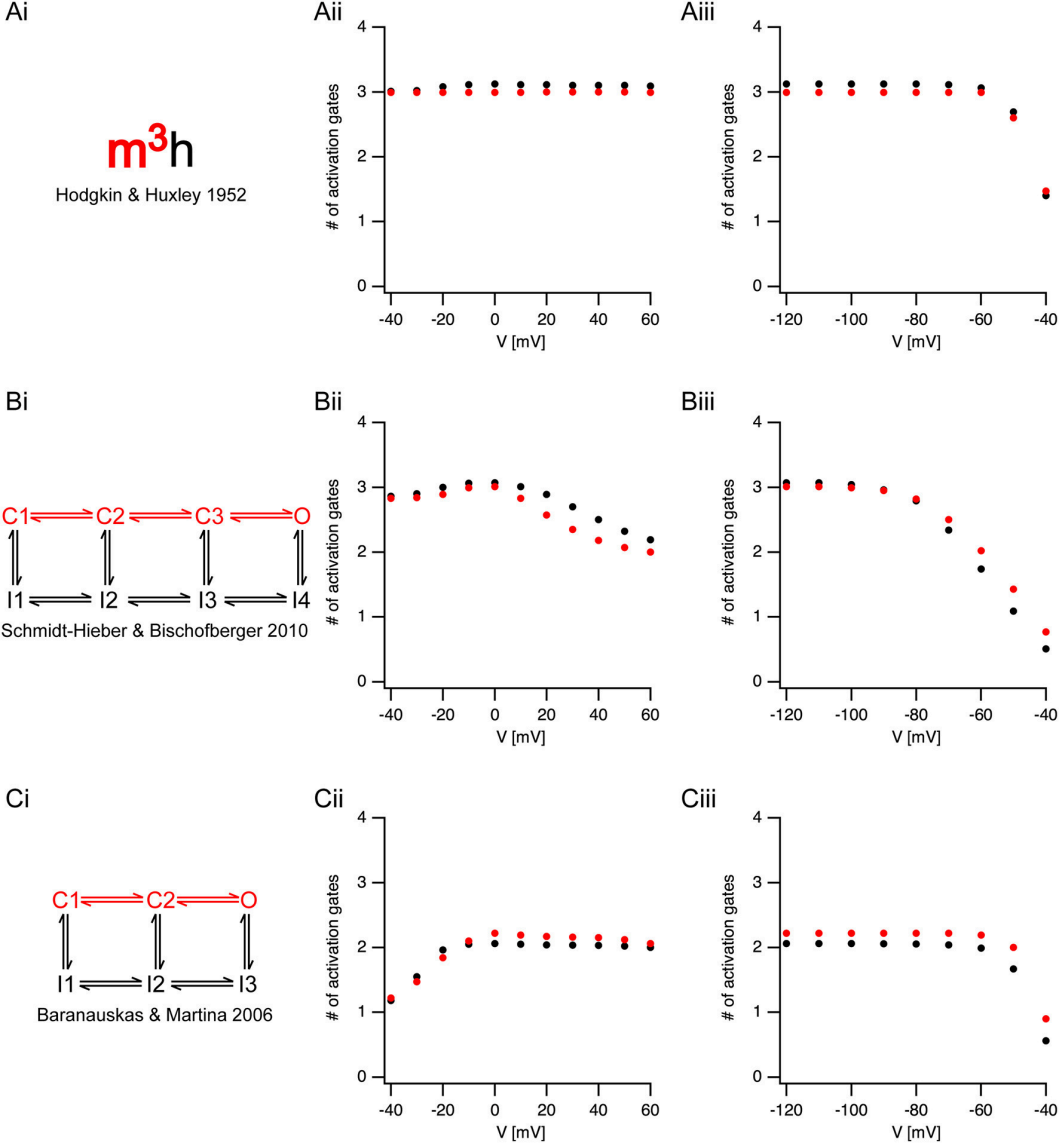
Estimating activation sigmoidicity in models of voltage-gated sodium channel. **(Ai,Bi,Ci)** A schematic representation of the model. The pertinent part of the model for activation appears in red. The source of each model is indicated below each scheme. **(Aii,Bii,Cii)**, The apparent number of activation gates, estimated by fitting Equation (3) to the initial onset of the current, plotted as a function of the potential in an activation protocol (the pre-pulse was 150 ms to −120 mV followed by a step to the test potential). The black circles are the values obtained from currents simulated using the full model. The red circles are the values obtained from currents simulated using only the activation part of the model. **(Aiii,Biii,Ciii)**, The apparent number of activation gates, estimated by fitting Equation (3) to the initial onset of the current, plotted as a function of the potential in the inactivation protocol (the pre-pulse varied from −120 mV in steps of 10 mV followed by a step to the 0 mV). Color coding is identical to that of **(Aii,Bii,Cii)**.

The analysis of sigmoidicity using Equation (3) has never been carried out on currents from voltage-gated sodium channels. To test this procedure, we applied it to three known models of voltage-gated sodium channels (Figure 4). It was simple to predict that the sigmoidicity of the Hodgkin and Huxley (Hodgkin and Huxley, 1952) model (Figure 4Ai) would be constant and equal to three. In fact, throughout the voltage range of channel activation our analysis of the initial current onset produced this expected result (Figure 4Aii). Fitting Equation (3) to the current onset of the inactivation protocol produced the expected result (*N* = 3) whereas the pre-pulse potential was below the activation threshold of the conductance (Figure 4Aiii). Above-activation threshold lower values of N were obtained.

Fitting Equation (3) up to 50% of the maximal current amplitude was performed under the assumption that the fit was not distorted by inactivation kinetics. To test this assumption, we removed the inactivation from the Hodgkin and Huxley model, and simulated non-inactivating currents. Fitting the onset of these currents to Equation (3) produced almost identical results to those obtained when fitting the full model (red markers in Figure 4). This verified that our assumption regarding the negligible effect of inactivation on the onset analysis was justified.

Next, we applied the onset analysis to two Markov models of voltage-gated sodium channels from the central nervous system. The model proposed by Schmidt-Hieber and Bischofberger (Schmidt-Hieber and Bischofberger, 2010) for hippocampal granule cells consists of three closed states (Figure 4Bi). We thus predicted that the onset analysis for some of the traces would be *N* = 3. This turned out to be the case for both the activation (Figure 4Bii) and inactivation (Figure 4Biii). However, unlike the Hodgkin and Huxley model, the number of gates predicted for the Schmidt-Hieber and Bischofberger model varied as a function of voltage as the occupancy of the states in the Markov model shifted from the first closed state all the way to the open state. This is very clear from Figure 4Biii that depicts the dependence of the sigmoidicity on the pre-pulse potential. As the pre-potential increased, the channel occupied closed states closer to the open state. Upon activation, the current trace activated with a shorter delay, thus leading to a smaller value of N when fitted to Equation (3). Consistent with the Schmidt-Hieber and Bischofberger model, fitting the Baranauskas and Martina (Baranauskas and Martina, 2006) model of cortical neurons (Figure 4Ci) to Equation (3) provided an approximate value of *N* = 2 for most potentials. Deviations from this value either in the activation (Figure 4Cii) or inactivation (Figure 4Ciii) protocols could easily be accounted for by the relative occupancy of the model in the closed states along with the rate of transition from the last closed state to the open state. To further test this assumption regarding the negligible influence of the inactivation kinetics on the curve fit we deleted the inactivation from the two Markov models and repeated the analysis only using the linear model containing closed states leading to the open state (marked in red in Figures 4Bi,Ci). These simulations generated values for N that were very similar to the full models (red symbols in Figure 4) further justifying the assumption we made in the analysis of the experimental traces (Figures 2, 3).

## Discussion

Here we showed that the activation of voltage-gated sodium channels in layer 5 pyramidal neurons from the rat neocortex does not follow traditional Hodgkin and Huxley kinetics. In addition, much of the channel voltage-dependence could be attributed to rapid closed-closed transitions that led to substantial onset latency reminiscent of the Cole-Moore effect observed in voltage-gated potassium conductances (Cole and Moore, 1960). Thus, the activation of the channels was state dependent and displayed a distinct delay if activated from hyperpolarized potentials, whereas there was almost immediate activation if activated from potentials closer to the action potential threshold.

The consequences of this state dependent activation to action potential firing are straightforward. We observed that the sigmoidicity of the sodium current increased as the holding potential was more negative (Figure 3). It is important to note that the increase in sigmoidicity was correlated to the increase in the delay before onset of channel activation. Given that the resting membrane potential of L5 pyramidal neurons ranged roughly from −70 to −60 mV, our crude analysis predicted that stepping from these potentials to above the channel activation threshold should generate a current displaying kinetics obeying ~4 activation gates. However, the physiological changes to the membrane potential are slower than the standard voltage-clamp step since they are limited by the membrane time constant. Thus, during synaptic integration changes to closed state occupancy of the sodium channel will clearly be faster than membrane potential changes. Consequently, as the membrane potential approaches the action potential threshold the sodium channel is likely to activate almost without delay.

The Cole and Moore (1960) study of the voltage-gated potassium channel of the giant squid axon showed that the holding potential could modulate the latency before the onset of activation. Hyperpolarization of the membrane potential increased the latency and the steepness of the current during activation (Cole and Moore, 1960). They reported that these effects increased the apparent number of activation gates in the potassium channel from 4 to 25. This “Cole-Moore” effect has also been observed in the shaker potassium channel (Hoshi et al., 1994; Zagotta et al., 1994a,b), the large conductance calcium activated potassium channel (Horrigan and Aldrich, 1999; Horrigan et al., 1999), and in the voltage-gated sodium channel in the squid giant axon (Keynes and Rojas, 1976). More recently, delayed activation has been observed in voltage-gated sodium channels in the rat central nervous system (Martina and Jonas, 1997; Baranauskas and Martina, 2006; Schmidt-Hieber and Bischofberger, 2010).

Of the studies reporting an activation delay in the rat central nervous system, two stand out since they present somewhat conflicting results. Baranauskas and Matina (Baranauskas and Martina, 2006) recorded voltage-gated current from several types of neurons in the rat CNS. They reported, in apparent contradiction to our results, that voltage-gated channels activate without a delay. Contrary to this study, Schmidt-Hieber and Bischofberger (Schmidt-Hieber and Bischofberger, 2010) reported that voltage-gated sodium channels display a distinct, voltage-dependent, activation delay in hippocampal granule cells. The discrepancy between these studies can be reconciled in view of the data we present here. Most of the recordings made by Baranauskas and Matina were carried out from a holding potential of −70 mV. Conversely, Schmidt-Hieber and Bischofberger applied a 50 ms pre-pulse to −120 mV to remove inactivation. This difference in the initial conditions of the experiments changes the occupancy of the closed states of the channels. Holding at −70 mV may be regarded as a more physiological initial condition which causes the channel to occupy a closed state close to the open state. The downside of this holding potential is of course the considerable inactivation that reduces the total current. The model presented by Baranauskas and Matina (Figure 4Ci), in which a single transition from the closed to the open state determines the activation kinetics of sodium currents, clearly captures some of the activation kinetics of the voltage-gated channel. The model presented by Schmidt-Hieber and Bischofberger (Figure 4Bi) that considers the voltage-dependence of the initial delay, contains an additional closed state. As the membrane potential approaches threshold, this closed state is largely unoccupied, which makes this model more similar to the Baranauskas and Matina model.

The role of the mechanism proposed here in action potential generation in L5 pyramidal neurons is probably part of a more complex mechanism. The action potential is known to be generated at the distal end of the axon initial segment (Palmer and Stuart, 2006; Hu et al., 2009; Popovic et al., 2011), though under some conditions it is generated at the first Ranvier node. That said, several studies have clearly shown complex crosstalk between the initiation zone and the soma (Khaliq and Raman, 2006; Palmer and Stuart, 2006; Foust et al., 2010; Telenczuk et al., 2017). The complexity of the axon initial segment derives from the composition and distribution of its voltage-gated ion channels, especially the voltage-gated sodium ion channels. Recently it was shown that the patch-clamp technique is probably inappropriate for determining the density of ion channels in the axon initial segment because of the ways in which these channels are anchored in the cytoskeleton with Ankyrin-G, which suggests, as models of the action potential have predicted, that the sodium channel density is high in the axon initial segment (Kole et al., 2008). Converging findings from labeling studies (Lorincz and Nusser, 2008; Royeck et al., 2008) and sodium imaging (Palmer and Stuart, 2006; Fleidervish et al., 2010; Baranauskas et al., 2013) suggest that the sodium channel density in the axon initial segment may be substantially larger than the soma, roughly ~4,000 pS/μm^2^. Using electrophysiology and immunohistochemistry, it was elegantly demonstrated that the axon initial segment has two different voltage-gated sodium channels, Nav1.2 and Nav1.6, and that these channels have graded distributions along the membrane (Hu et al., 2009); Nav1.6 has a higher density in the distal part of the initial segment while Nav1.2 dominates the soma, and this anatomical complexity explains much of the axon initial segment's physiological complexity. In the current study, we recorded from nucleated patches extracted from the soma. Thus, our conclusions regarding the role of the delayed activation of the voltage-gated sodium channel, while logical, should be probed by recording the activity of NaV1.6 in axonal patches or expression systems.

Regardless of the role played by the activation delay in action potential firing, our results clearly indicate a need to re-evaluate the application of the Hodgkin and Huxley formalism to modeling the role of ions in neuronal excitability and the value of replacing it with Markov chain models. Like all models, the Hodgkin and Huxley model approximates reality. As such, it performs well under certain conditions and fails in others. As noted above (Cole and Moore, 1960; Moore, 2015), Hodgkin and Huxley carried out most of their recordings from a holding potential of −70 mV. Thus, it is not surprising that they obtained a relatively short delay, leading to the claim of three activation gates for the sodium channels and four activation gates for the potassium channels. However, this selection of activation gates eliminated a dynamic aspect of the channel from the model. This can clearly be seen in Figure 4Aii in which the sigmoidicity does not change as a function of voltage. The large sigmoidicity we report here indicates that the full model for the voltage-gated channel should probably contain at least 7–8 closed states. The full scope of this model, which should also include the fast and slow inactivation processes, has yet to be determined.

## Author contributions

MA Designed research, Recorded data, analyzed data, wrote paper. TB Analysed data, wrote paper. AL Wrote paper. AK Designed research, analyzed data, wrote paper.

### Conflict of interest statement

The authors declare that the research was conducted in the absence of any commercial or financial relationships that could be construed as a potential conflict of interest.

## References

[B1] Almog M. Korngreen A. (2009). Characterization of voltage-gated Ca^(2+)^ conductances in layer 5 neocortical pyramidal neurons from rats. PLoS ONE 4:e4841. 10.1371/journal.pone.0004841 19337371 PMC2659773

[B2] Armstrong C. M. Bezanilla F. (1977). Inactivation of the sodium channel. II. Gating Curr. Exp. J. Gen. Physiol. 70, 567–590. 10.1085/jgp.70.5.567 PMC2228472 591912

[B3] Astman N. Gutnick M. J. Fleidervish I. A. (2006). Persistent sodium current in layer 5 neocortical neurons is primarily generated in the proximal axon. J. Neurosci. 26, 3465–3473. 10.1523/JNEUROSCI.4907-05.2006 16571753 PMC6673860

[B4] Baranauskas G. David Y. Fleidervish I. A. (2013). Spatial mismatch between the Na^+^ flux and spike initiation in axon initial segment. Proc. Natl. Acad. Sci. U.S.A. 110, 4051–4056. 10.1073/pnas.1215125110 23341597 PMC3593864

[B5] Baranauskas G. Martina M. (2006). Sodium currents activate without a Hodgkin-and-Huxley-type delay in central mammalian neurons. J. Neurosci. 26, 671–684. 10.1523/JNEUROSCI.2283-05.2006 16407565 PMC6674426

[B6] Bezanilla F. Armstrong C. M. (1977). Inactivation of the sodium channel. I. sodium current experiments. J. Gen. Physiol. 70, 549–566. 10.1085/jgp.70.5.549 591911 PMC2228478

[B7] Brumberg J. C. Nowak L. G. McCormick D. A. (2000). Ionic mechanisms underlying repetitive high-frequency burst firing in supragranular cortical neurons. J. Neurosci. 20, 4829–4843. 10.1523/JNEUROSCI.20-13-04829.2000 10864940 PMC6772270

[B8] Cole K. S. Moore J. W. (1960). Potassium ion current in the squid giant axon: dynamic characteristic. Biophys. J. 1, 1–14. 10.1016/S0006-3495(60)86871-3 13694549 PMC1366308

[B9] Fleidervish I. A. Friedman A. Gutnick M. J. (1996). Slow inactivation of Na^+^ current and slow cumulative spike adaptation in mouse and guinea-pig neocortical neurones in slices. J. Physiol. 493, 83–97. 10.1113/jphysiol.1996.sp021366 8735696 PMC1158952

[B10] Fleidervish I. A. Gutnick M. J. (1996). Kinetics of slow inactivation of persistent sodium current in layer V neurons of mouse neocortical slices. J. Neurophysiol. 76, 2125–2130. 10.1152/jn.1996.76.3.2125 8890326

[B11] Fleidervish I. A. Lasser-Ross N. Gutnick M. J. Ross W. N. (2010). Na^+^ imaging reveals little difference in action potential-evoked Na^+^ influx between axon and soma. Nat. Neurosci. 13, 852–860. 10.1038/nn.2574 20543843 PMC3102307

[B12] Foust A. Popovic M. Zecevic D. McCormick D. A. (2010). Action potentials initiate in the axon initial segment and propagate through axon collaterals reliably in cerebellar Purkinje neurons. J. Neurosci. 30, 6891–6902. 10.1523/JNEUROSCI.0552-10.2010 20484631 PMC2990270

[B13] Hamill O. P. Marty A. Neher E. Sakmann B. Sigworth F. J. (1981). Improved patch-clamp techniques for high-resolution current recording from cells and cell-free membrane patches. Pflugers Arch. 391, 85–100. 10.1007/BF00656997 6270629

[B14] Hines M. L. Carnevale N. T. (1997). The NEURON simulation environment. Neural Comput. 9, 1179–1209. 10.1162/neco.1997.9.6.1179 9248061

[B15] Hines M. L. Carnevale N. T. (2000). Expanding NEURON's repertoire of mechanisms with NMODL. Neural Comput. 12, 995–1007. 10.1162/089976600300015475 10905805

[B16] Hodgkin A. L. Huxley A. F. (1952). A quantitative description of membrane current and its application to conduction and excitation in nerve. J. Physiol. 117, 500–544. 10.1113/jphysiol.1952.sp004764 12991237 PMC1392413

[B17] Horrigan F. T. Aldrich R. W. (1999). Allosteric voltage gating of potassium channels II. Mslo channel gating charge movement in the absence of Ca^(2+)^. J .Gen. Physiol. 114, 305–336. 10.1085/jgp.114.2.305 10436004 PMC2230644

[B18] Horrigan F. T. Cui J. Aldrich R. W. (1999). Allosteric voltage gating of potassium channels I. Mslo ionic currents in the absence of Ca^(2+)^. J. Gen. Physiol. 114, 277–304. 10.1085/jgp.114.2.277 10436003 PMC2230643

[B19] Hoshi T. Zagotta W. N. Aldrich R. W. (1994). Shaker potassium channel gating. I: transitions near the open state. J. Gen. Physiol. 103, 249–278. 10.1085/jgp.103.2.249 8189206 PMC2216835

[B20] Hu W. Tian C. Li T. Yang M. Hou H. Shu Y. (2009). Distinct contributions of Na(v)1.6 and Na(v)1.2 in action potential initiation and backpropagation. Nat. Neurosci. 12, 996–1002. 10.1038/nn.2359 19633666

[B21] Keynes R. D. Rojas E. (1976). The temporal and steady-state relationships between activation of the sodium conductance and movement of the gating particles in the squid giant axon. J. Physiol. 255, 157–189. 10.1113/jphysiol.1976.sp011274 1255514 PMC1309239

[B22] Khaliq Z. M. Raman I. M. (2006). Relative contributions of axonal and somatic Na^+^ channels to action potential initiation in cerebellar Purkinje neurons. J. Neurosci. 26, 1935–1944. 10.1523/JNEUROSCI.4664-05.2006 16481425 PMC6674931

[B23] Kole M. H. Ilschner S. U. Kampa B. M. Williams S. R. Ruben P. C. Stuart G. J. (2008). Action potential generation requires a high sodium channel density in the axon initial segment. Nat. Neurosci. 11, 178–186. 10.1038/nn2040 18204443

[B24] Kole M. H. Stuart G. J. (2008). Is action potential threshold lowest in the axon? Nat. Neurosci. 11, 1253–1255. 10.1038/nn.2203 18836442

[B25] Korngreen A. Sakmann B. (2000). Voltage-gated K^+^ channels in layer 5 neocortical pyramidal neurones from young rats: subtypes and gradients. J. Physiol. 523, 621–639. 10.1111/j.1469-7793.2000.00621.x 10856117 PMC2269970

[B26] Lewis A. H. Raman I. M. (2014). Resurgent current of voltage-gated Na^+^ channels. J. Physiol. 592, 4825–4838. 10.1113/jphysiol.2014.277582 25172941 PMC4259529

[B27] Lorincz A. Nusser Z. (2008). Cell-type-dependent molecular composition of the axon initial segment. J. Neurosci. 28, 14329–14340. 10.1523/JNEUROSCI.4833-08.2008 19118165 PMC2628579

[B28] Martina M. Jonas P. (1997). Functional differences in Na^+^ channel gating between fast-spiking interneurones and principal neurones of rat hippocampus. J. Physiol. 505, 593–603. 10.1111/j.1469-7793.1997.593ba.x 9457638 PMC1160038

[B29] Milescu L. S. Akk G. Sachs F. (2005). Maximum likelihood estimation of ion channel kinetics from macroscopic currents. Biophys. J. 88, 2494–2515. 10.1529/biophysj.104.053256 15681642 PMC1305347

[B30] Moore J. W. (2015). Enhancing the hodgkin-huxley equations: simulations based on the first publication in the biophysical journal. Biophys. J. 109, 1317–1320. 10.1016/j.bpj.2015.08.008 26445431 PMC4601065

[B31] Palmer L. M. Stuart G. J. (2006). Site of action potential initiation in layer 5 pyramidal neurons. J. Neurosci. 26, 1854–1863. 10.1523/JNEUROSCI.4812-05.2006 16467534 PMC6793621

[B32] Patlak J. (1991). Molecular kinetics of voltage-dependent Na^+^ channels. Physiol. Rev. 71, 1047–1080. 10.1152/physrev.1991.71.4.1047 1656476

[B33] Popovic M. A. Foust A. J. McCormick D. A. Zecevic D. (2011). The spatio-temporal characteristics of action potential initiation in layer 5 pyramidal neurons: a voltage imaging study. J. Physiol. 589, 4167–4187. 10.1113/jphysiol.2011.209015 21669974 PMC3180577

[B34] Raman I. M. Bean B. P. (1997). Resurgent sodium current and action potential formation in dissociated cerebellar Purkinje neurons. J. Neurosci. 17, 4517–4526. 10.1523/JNEUROSCI.17-12-04517.1997 9169512 PMC6573347

[B35] Raman I. M. Bean B. P. (2001). Inactivation and recovery of sodium currents in cerebellar Purkinje neurons: evidence for two mechanisms. Biophys. J. 80, 729–737. 10.1016/S0006-3495(01)76052-3 11159440 PMC1301271

[B36] Raman I. M. Sprunger L. K. Meisler M. H. Bean B. P. (1997). Altered subthreshold sodium currents and disrupted firing patterns in Purkinje neurons of Scn8a mutant mice. Neuron 19, 881–891. 10.1016/S0896-6273(00)80969-1 9354334

[B37] Royeck M. Horstmann M. T. Remy S. Reitze M. Yaari Y. Beck H. (2008). Role of axonal NaV1.6 sodium channels in action potential initiation of CA1 pyramidal neurons. J. Neurophysiol. 100, 2361–2380. 10.1152/jn.90332.2008 18650312

[B38] Sakmann B. Neher E. (1995). Single Channel Recording. New York, NY: Plenum press.

[B39] Sather W. Dieudonne S. MacDonald J. F. Ascher P. (1992). Activation and desensitization of N-methyl-D-aspartate receptors in nucleated outside-out patches from mouse neurones. J. Physiol. 450, 643–672. 10.1113/jphysiol.1992.sp019148 1359126 PMC1176143

[B40] Schmidt-Hieber C. Bischofberger J. (2010). Fast sodium channel gating supports localized and efficient axonal action potential initiation. J. Neurosci. 30, 10233–10242. 10.1523/JNEUROSCI.6335-09.2010 20668206 PMC6633381

[B41] Schoppa N. E. Sigworth F. J. (1998a). Activation of Shaker potassium channels. III. an activation gating model for wild-type and V2 mutant channels. J. Gen. Physiol. 111, 313–342. 10.1085/jgp.111.2.313 9450946 PMC2222769

[B42] Schoppa N. E. Sigworth F. J. (1998b). Activation of shaker potassium channels. I. characterization of voltage-dependent transitions. J. Gen. Physiol. 111, 271–294. 10.1085/jgp.111.2.271 9450944 PMC2222764

[B43] Stuart G. J. Dodt H.-U. Sakmann B. (1993). Patch-clamp recordings from the soma and dendrites of neurons in brain slices using infrared video microscopy. Pflüg Arch. Eur. J. Physiol. 423, 511–518. 10.1007/BF00374949 8351200

[B44] Stuart G. Sakmann B. (1995). Amplification of EPSPs by axosomatic sodium channels in neocortical pyramidal neurons. Neuron 15, 1065–1076. 10.1016/0896-6273(95)90095-0 7576650

[B45] Telenczuk M. Fontaine B. Brette R. (2017). The basis of sharp spike onset in standard biophysical models. PLoS ONE 12:e0175362. 10.1371/journal.pone.0175362 28441389 PMC5404793

[B46] Zagotta W. N. Hoshi T. Aldrich R. W. (1994a). Shaker potassium channel gating. III: evaluation of kinetic models for activation. J. Gen. Physiol. 103, 321–362. 10.1085/jgp.103.2.321 8189208 PMC2216839

[B47] Zagotta W. N. Hoshi T. Dittman J. Aldrich R. W. (1994b). Shaker potassium channel gating. II: transitions in the activation pathway. J. Gen. Physiol. 103, 279–319. 10.1085/jgp.103.2.279 8189207 PMC2216838

